# Estimating Three-Dimensional Orientation of Human Body Parts by Inertial/Magnetic Sensing

**DOI:** 10.3390/s110201489

**Published:** 2011-01-26

**Authors:** Angelo Maria Sabatini

**Affiliations:** The BioRobotics Institute, Scuola Superiore Sant’Anna, Piazza Martiri della Libertà 33, 56124 Pisa, Italy; E-Mail: sabatini@sssup.it; Tel.: +39-050-883-415; Fax: +39-050-883-101

**Keywords:** human body motion tracking, inertial/magnetic sensing, strap-down inertial navigation, sensor fusion, Kalman filtering, quaternion

## Abstract

User-worn sensing units composed of inertial and magnetic sensors are becoming increasingly popular in various domains, including biomedical engineering, robotics, virtual reality, where they can also be applied for real-time tracking of the orientation of human body parts in the three-dimensional (3D) space. Although they are a promising choice as wearable sensors under many respects, the inertial and magnetic sensors currently in use offer measuring performance that are critical in order to achieve and maintain accurate 3D-orientation estimates, anytime and anywhere. This paper reviews the main sensor fusion and filtering techniques proposed for accurate inertial/magnetic orientation tracking of human body parts; it also gives useful recipes for their actual implementation.

## 1. Introduction

The problem of accurate tracking of the orientation (attitude) of rigid objects is important in several domains, among them navigation of man-made vehicles, e.g., air and spacecrafts, robotics and, of interest in this paper, ambulatory human movement analysis, which may include a range of interesting applications, from monitoring of activities of daily living (ADL) to virtual/augmented reality (VR/AR). Several technologies and approaches are available to produce motion tracking systems (trackers), which derive orientation estimates from electrical measurements of acoustic, inertial, magnetic, mechanical, optical and radio frequency sensors [1]. One increasingly popular approach is based on using inertial and magnetic sensors.

Several factors explain the popularity of inertial/magnetic sensing. Most current sensing approaches for motion tracking need the availability of external sources, e.g., cameras for optical trackers, ultrasonic/electromagnetic transmitters for acoustic/electromagnetic trackers. Usually, the sources can operate only over relatively short distances, which makes the trackers highly susceptible to interference and line-of-sight occlusion (shadowing): hence, proper functioning of these trackers is only possible within carefully controlled experimental setups (motion analysis laboratories). This fact precludes, for instance, the quantitative assessment of the behaviour of a human subject in unrestrained conditions. Conversely, inertial sensors are completely self-contained (sourceless), since they measure physical quantities, such as linear acceleration and angular velocity, which are related to the motion of the objects where the sensors are fixed; moreover, although magnetic sensing is externally referenced, the ubiquitous presence of a magnetic field on earth makes the magnetic source available almost everywhere. Recent technological advances in the field of micro-electro-mechanical systems (MEMS) have made it possible to manufacture inertial sensors that are relatively low cost, highly miniaturized and with limited power consumption. Inertial/magnetic sensors can therefore be considered the most valuable opportunity to monitor the ADLs of a human subject outside specialized laboratories, and over possibly extended periods of time. However, the measuring accuracies of MEMS inertial sensors are still largely inferior to those of the sensors used, e.g., in inertial navigation systems (INSs) for aeronautical and military applications [2]. Hence, it becomes of the utmost importance concentrating on the development of efficient filtering algorithms for applications of these sensors, together with modern solid-state magnetic sensors, in human body motion capture.

Accurate estimates of the three-dimensional (3D) orientation of a rigid body by inertial/magnetic sensing require that the complementary properties of gyros, accelerometers and magnetic sensors are purposefully exploited [3]. The orientation can be computed by time-integrating, from known initial conditions, the signals from a triad of mutually orthogonal uni-axial gyros (tri-axial gyro), which is prone to errors that grow unbounded over time, due to low-frequency gyro bias drifts; on the other hand, gyros help achieving accurate orientation estimates for highly dynamic motions. A tri-axial accelerometer is capable of providing drift-free inclination estimates by sensing the gravity vector. It can be used alone or, when heading estimation is also needed, together with a tri-axial magnetic sensor, giving rise to a sensing unit that is referred to in the following as a gyro-free aiding sensor system. Serious limitations affect the operation of a gyro-free aiding sensor system. First, the difficulty of correctly interpreting the acceleration signals, when the component due to the gravity field (vertical reference) coexists with the component related to the motion of the object. Hence, the vertical reference is reliable only for static or slowly moving objects [4]. Second, nearby ferromagnetic materials are critically disturbing sources when attempts are made to interpret the signals from a tri-axial magnetic sensor as the horizontal reference; this problem becomes especially acute within man-made indoor environments [5,6]. Sensor fusion techniques are needed in order that the gyro-free aiding sensor system allows bounding the gyro bias drift errors; in turn, the gyros can be used to smooth the orientation estimates provided by the gyro-free aiding sensor system. Ideally, the filtering algorithm would be also capable of estimating gyro biases, as well as biases in the sensors of the gyro-free aiding sensor system.

The main purpose of this paper is to review important methods for the design of these filtering algorithms. Section 2 surveys main sensing approaches proposed by researchers active in biomedical engineering, biomechanics and related fields. Section 3 provides the reader with background information about mathematical methods for representing the orientation; the kinematic equations of a rigid body and simple numerical methods for their solution are also briefly discussed. Section 4 reviews the main deterministic and stochastic algorithms for estimating the orientation, with particular emphasis on vector matching, linear Kalman Filters (KFs) and the their extended (EKF) version, suited for nonlinear models; KFs and EKFs are presented here as special cases of Bayesian filters. Section 5 discusses the modelling issues behind the implementation of a state-of-the-art EKF, and presents a worked-out example related to a head motion tracking trial. The paper concludes with Section 6.

## 2. Sensing Approaches

A number of pioneering contributions in the 60–70s suggested reconstructing the field of motion of a rigid body, in terms of both position and orientation (pose), by sampling acceleration values in several suitably selected points of it. The rotation of a rigid body with one point fixed requires a minimum of three acceleration measurements; at least, three additional acceleration measurements are needed to resolve the motion of the fixed point in the 3D-space [7]. Sensor systems composed of several accelerometers, suitably arranged in uni-axial, bi-axial, tri-axial clusters, were proposed in [8], for applications mainly in the field of impact biomechanics. It was proven that, in the presence of small experimental errors, numerical drifts in the pose estimation make systems with six accelerometers inherently unstable; even systems with nine accelerometers exhibit critical performance degradation. The six-nine sensor configurations were analyzed in depth in [9], to conclude that severe restrictions exist in the time duration over which motion tracking is feasible by accelerometry methods in routine biomechanical applications. The problem with these configurations is that the angular velocity has to be estimated by time-integrating noisy measured angular accelerations, which restricts the time horizon for accurate motion tracking. More redundancy is necessary to achieve stability and tolerance to positioning/alignment errors of the accelerometers, provided that suitable calibration procedures are also implemented. Since angular velocity is not determined by time-integration anymore, kinematically redundant systems with twelve accelerometers are reported to yield promising results [10].

During the 80–90s, the use of accelerometers in biomechanics was promoted mainly in the clinical assessment of gait: under the simplifying assumption of a gait motion planar model, a minimal configuration set composed of two leg-mounted single-axis accelerometers with parallel sensitive axes would suffice to determine the angular acceleration of the leg. In the attempt to circumvent the problem of numerical integration drift, pairs of accelerometers on each segment were used to resolve the relative angle between two segments, namely the joint angle, without time-integration [11]. These accelerometer-based angle sensors were discussed in [12], where the most important error sources were analyzed in detail, namely not fulfilling the gait motion planar model and the rigid-body condition by external fixation of body-mounted sensors. The potential of accelerometers as sensors that are capable of measuring the inclination of human body parts in gait analysis has been emphasized in later research, e.g., [13]. Very interesting is the work in [14], where the authors used one tri-axial accelerometer to measure inclination during dynamic tasks without requiring additional sensors. A KF-based algorithm was designed to estimate the different acceleration components, namely gravity and inertial acceleration, plus the accelerometer bias, using a simple model of the human motion dynamics. Since the procedure of bias compensation in the KF-algorithm works only in the direction of gravity, the bias estimate in all measurement directions turns out to be reasonably accurate only when the accelerometer is rotated over large angles. In any case, the method was shown to outperform the method based on low-pass filtering the accelerometer signals [4,15], especially as the speed of motion increased.

Toward the end of the 90s and in the early years of this century, the emergence on the consumer market of miniaturized MEMS gyros, with good metrological specifications and low cost, opened a new way to think about the role of inertial sensing in human body motion tracking and analysis [15,16]. At the time when solid-state magnetic sensors also found their way on the consumer market [17], miniaturized, fully integrated inertial/magnetic measurement units (IMMUs) became finally available for use in strap-down INSs. In a strap-down INS the signals produced by the inertial/magnetic sensors are resolved mathematically in a computer, prior to the calculation of navigational information [18]. Using a computer to resolve the inertial/magnetic data reduces the mechanical complexity of an INS, as it is implemented in the classical applications of inertial navigation technology, *i.e.*, stable platform technique, thus decreasing the cost and size of the system and consequently increasing its reliability. The processing speeds of modern computers and microcomputers and their low-cost allow conceiving efficient implementations of wearable strap-down INSs for human body motion capture.

However, it is critical to achieve high accuracy in pose determination by strap-down INSs that incorporate low-cost inertial/magnetic sensors, since their stand-alone accuracy and run-to-run stability are poor. Different applications may involve different accuracy requirements relative to the duration of each observation run: in the absence of special precautions, the requirements of human motion tracking applications are shown to be violated when the duration of the observation run exceeds just several seconds [3]. Nonetheless, during the late 90s inertial tracking with automatic drift correction proved to be a highly successful technique for challenging applications in VR/AR, offering low jitter, fast response, increased range, and greatly reduced problems due to interference and shadowing [19]. InterSense Inc., Billerica, MA, USA pioneered the commercial development of trackers based on miniature MEMS inertial sensors. At the time being, few other companies are marketing IMMUs, among them: Xsens Technologies B.V. (Enschede, The Netherlands); and MicroStrain Inc. (Williston, VT, USA). Several research groups are now active in exploiting them for biomechanical applications, with concentration on the design of filtering algorithms, e.g., [20–25].

Besides being important *per se*, estimating the orientation is fundamental in the strap-down approach to position estimation: in fact, the orientation solution allows the gravity to be cancelled from the acceleration signals, in order that the inertial acceleration is double-integrated for position estimation (gravity compensation) [3]. If the gravity compensation is not carried out properly, the orientation errors add to the positioning errors, to yield a devastating growth of positioning errors that are proportional to the cube of the system’s operation time [26]. There appear to be different means to deal with these problems, e.g., using externally referenced aids, such as Global Positioning System (GPS), and carry out the integration process underlying the combined use of GPS and INS technologies using KF techniques, e.g., [27,28]. Another approach is to exploit idiosyncrasies of the human motion dynamics by designing algorithms that can keep the drift rate low [29]. The problem of position determination is not addressed in this paper.

## 3. Representation and Determination of Orientation: Mathematical Review

### 3.1. Representation of Orientation

**Figure 1.**
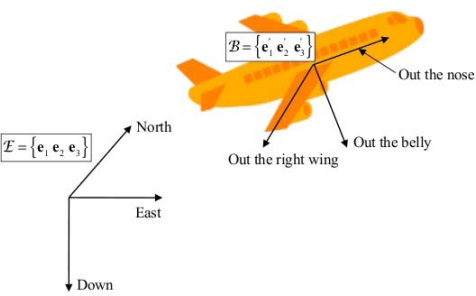
Earth-fixed frame and body-fixed frame on a toy aircraft.

For motion on or near the earth surface, at speeds far below orbital velocity, it is convenient to describe the orientation of a rigid body using two coordinate systems: the earth-fixed coordinate system, specified by the right-handed orthonormal basis E = {**e**_1_ **e**_2_ **e**_3_}, whose coordinate axes are directed in the local north, east and down directions (NED)—for all practical purposes, an inertial coordinate system; the non-inertial coordinate system, aka body-fixed coordinate system, specified by the right-handed orthonormal basis $B=\left\{e_{1}^{\prime }\;e_{2}^{\prime }\;e_{3}^{\prime }\right\}$, whose coordinate axes are conventionally named “out the nose”, “out the right side” and “out the belly” in the aeronautics jargon (Figure 1).

We recall that an orthonormal basis T = {**i j k**} is said to be right-handed if it satisfies:

(1) i×j=k,    j×k=i,    k×i=j .

where the symbol × denotes the standard vector cross product.

An arbitrary vector **x** in the 3D space can be written in the equivalent forms:

(2) $$\begin{array}{l} x=x_{1}\;e_{1}+x_{2}\;e_{2}+x_{3}\;e_{3}\\ x=x_{1}^{\prime }e_{1}^{\prime }+x_{2}^{\prime }\;e_{2}^{\prime }+x_{3}^{\prime }\;e_{3}^{\prime }. \end{array}$$

The vector **x** can therefore be represented in terms of the coordinates (or components) with respect to either basis:

(3) $$\begin{array}{l} x_{E}={\left[x_{1}\;x_{2}\;x_{3}\right]}^{T}\\ x_{B}={\left[x_{1}^{\prime }\;x_{2}^{\prime }\;x_{3}^{\prime }\right]}^{T}. \end{array}$$

The subscripts E, B indicate which basis is used for representing the vector **x**. The representations in (3) are related to one another as follows:

(4) $$x_{B}={}_{E}^{B}C\;x_{E}.$$

The columns of the direction cosine matrix (DCM) ${}_{E}^{B}C$ are the representations of the **e***_i_*, *i* = 1,…,3 with respect to B, while its rows are the representations of the $e_{i}^{\prime }$, *i* = 1,…,3 with respect to E. The DCM, also called orientation (attitude) matrix, and its transpose allow therefore moving vector representations from (to) the earth-fixed frame to (from) the body-fixed frame, respectively. The orientation matrix is a 3 × 3 orthogonal matrix with unit determinant, which belongs to the three-dimensional special orthogonal group SO(3) of rotation matrices. Although the orientation matrix is the fundamental representation of the orientation, the orthogonality requirement forces six constraints on its nine elements, namely the column (row) vectors have unit norm and are mutually orthogonal, yielding that the special orthogonal group SO(3) of rotation matrices has dimension three.

**Figure 2.**
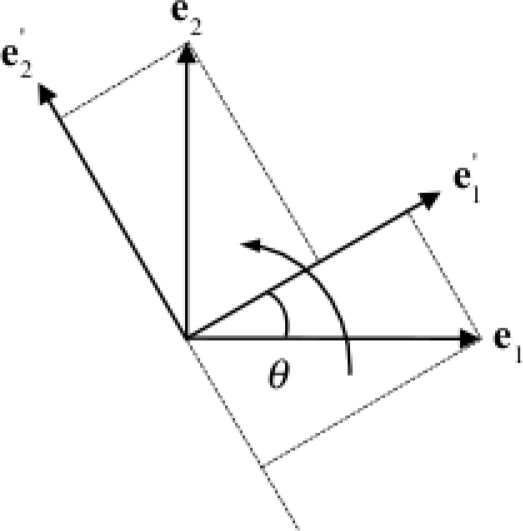
Rotation about the **e**_3_-axis through an angle *θ* (positive counter-clockwise).

Lower-dimensional parameterizations of orientation can be derived based on the following considerations [30]. As shown in Figure 2, a rotation about the **e**_3_-axis through an angle *θ* is expressed as:

(5) $$\{\begin{array}{l} e_{1}^{\prime }=\text{cos}\;\theta \;e_{1}+\text{sin}\;\theta \;e_{2}\\ e_{2}^{\prime }=-\text{sin}\;\theta \;e_{1}+\text{cos}\;\theta \;e_{2}\\ e_{3}^{\prime }=e_{3}. \end{array}$$

The resulting rotation matrix is:

(6) $$R\left(e_{3},\theta \right)=\left[\begin{array}{ccc} c_{\theta } & s_{\theta } & 0\\ -s_{\theta } & c_{\theta } & 0\\ 0 & 0 & 1 \end{array}\right].$$

where *c_θ_* and *s_θ_* are compact notation for cos*θ* and sin*θ*, respectively. By analogy with (6), the rotation matrices that describe rotations about the **e**_2_-axis and the **e**_1_-axis through an angle *θ* are:

(7) $$R\left(e_{2},\theta \right)=\left[\begin{array}{ccc} c_{\theta } & 0 & -s_{\theta }\\ 0 & 1 & 0\\ s_{\theta } & 0 & c_{\theta } \end{array}\right]\;\;\;\;R\left(e_{1},\theta \right)=\left[\begin{array}{ccc} 1 & 0 & 0\\ 0 & c_{\theta } & s_{\theta }\\ 0 & -s_{\theta } & c_{\theta } \end{array}\right].$$

We note that:

(8) $$\{\begin{array}{l} R\left(e_{3},\theta \right)\;e_{1}=c_{\theta }\;e_{1}-s_{\theta }\;e_{2}=c_{\theta }\;e_{1}-s_{\theta }\;e_{3}\times e_{1}\\ R\left(e_{3},\theta \right)\;e_{2}=c_{\theta }\;e_{2}+s_{\theta }\;e_{1}=c_{\theta }\;e_{2}-s_{\theta }\;e_{3}\times e_{2}\\ R\left(e_{3},\theta \right)\;e_{3}=\;e_{3}. \end{array}$$

For the vector cross product, an equivalent expression is:

(9) u×v=[u×] v,

where [**u** ×] is the skew-symmetric matrix:

(10) $$\left[u\times \right]=\left[\begin{array}{ccc} 0 & -u_{3} & u_{2}\\ u_{3} & 0 & -u_{1}\\ -u_{2} & u_{1} & 0 \end{array}\right].$$

**Figure 3.**
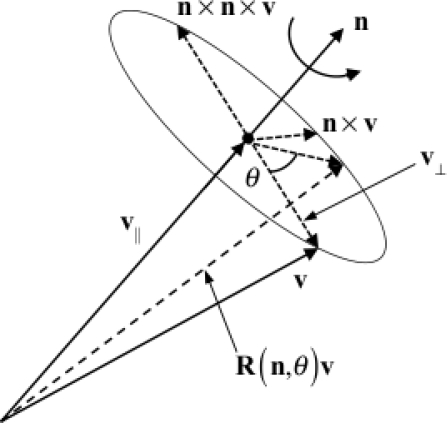
The vector **v** is rotated about the axis **n** (Euler axis) through an angle of rotation *θ*. Note that the rotated vector **R** (**n**, *θ*)**v** shares the parallel component with **v**.

Be **n** any unit column vector, and be **v**_⊥_ the projection of a column vector **v** onto the plane perpendicular to **n** (Figure 3). By analogy with (8) we can write:

(11) $$\{\begin{array}{l} R\left(n,\theta \right)\;v_{⊥}=c_{\theta }\;v_{⊥}-s_{\theta }\;\left[n\times \right]\;v\\ R\left(n,\theta \right)\;n=n. \end{array}$$

For arbitrary vectors **a**, **b** and **c** the Grassman identity yields:

(12) a×(b×c)=(a⋅c) b−(a⋅b) c.

The symbol □ denotes the standard vector dot product. Equation (12) allows deriving the general decomposition of **v** into components that are parallel (**v**_P_) and perpendicular (**v**_⊥_) to **n**:

(13) $$n\times \left(n\times v\right)=\left(n\cdot v\right)\;n-\left(n\cdot n\right)\;v\;\to \;v={\mathrm{nn}}^{T}v-{\left[n\times \right]}^{2}\;v=v_{P}+v_{⊥}.$$

In conclusion:

(14) $$R\left(n,\theta \right)\;v=R\left(n,\theta \right)\;\left(v_{P}+v_{⊥}\right)=v_{P}+c_{\theta }\;v_{⊥}-s_{\theta }\;\left[n\times \right]\;v_{⊥}.$$

It follows that two equivalent expressions of the rotation matrix are written (Euler’s formula):

(15) $$\{\begin{array}{l} R\left(n,\theta \right)=c_{\theta }\;I_{3}+\left(1-c_{\theta }\right)\;{\mathrm{nn}}^{T}-s_{\theta }\;\left[n\times \right]\\ R\left(n,\theta \right)=I_{3}-s_{\theta }\;\left[n\times \right]+\left(1-c_{\theta }\right)\;{\left[n\times \right]}^{\;2}. \end{array}$$

where **I***_n_* denotes the *n* × *n* identity matrix.

Euler’s theorem states that the most general motion of a rigid body with one point fixed is a rotation by an angle *θ* (rotation angle) about some axis **n** (rotation axis), yielding another representation of the orientation in terms of the rotation vector:

(16) θ=θ n.

All rotations can thus be mapped to points inside and on the surface of a sphere of radius *π* in rotation vector space (*θ* ∈]−*π*, *π*]). Since points at opposite ends of any diameter of the sphere represent the same orientation, the parameterization of orientation through the rotation vector is redundant, with four parameters and one constraint enforced on its norm. Moreover, no points of singularity exist in the rotation vector space.

The orientation of the body-fixed frame relative to the earth-fixed frame can also be described using the Euler angle formulation, namely in terms of three consecutive rotations through three body-referenced Euler angles [31]. Although, in principle, twelve possible ways exist to define three independent body-referenced Euler angles, just a subset of them have received attention; we discuss here the 3-2-1 rotation sequence, which is the one commonly adopted in the aeronautics community. The orientation of the body-fixed frame (nose-wing-belly) relative to the earth-fixed frame (NED) is described by performing the three rotations as follows. Start with a body-fixed frame in the reference orientation, *i.e.*, one in which all of its body-fixed axes are aligned with the corresponding earth axes; first, the body is rotated about the belly axis through an angle *ψ* usually called heading angle, or yaw (*ψ* ∈ ]−*π*, *π*]); second, the object is rotated about the wing axis through an angle ϑ (elevation angle, or pitch attitude) (ϑ ∈]−*π*/2, *π*/2]); third, the object is rotated about the nose axis through an angle *φ* (bank angle, or roll attitude), so as to match the body-fixed frame (*φ* ∈]−*π*, *π*]). We can then write:

(17) $$x_{B}=\left[\begin{array}{ccc} 1 & 0 & 0\\ 0 & c_{\varphi } & s_{\varphi }\\ 0 & -s_{\varphi } & c_{\varphi } \end{array}\right]\;\left[\begin{array}{ccc} c_{\vartheta } & 0 & -s_{\vartheta }\\ 0 & 1 & 0\\ s_{\vartheta } & 0 & c_{\vartheta } \end{array}\right]\;\left[\begin{array}{ccc} c_{\psi } & s_{\psi } & 0\\ -s_{\psi } & c_{\psi } & 0\\ 0 & 0 & 1 \end{array}\right]\;x_{E}.$$

The rotation matrix as a function of the three Euler angles is given by:

(18) $$R\left(\psi ,\vartheta ,\varphi \right)=[\begin{array}{ccc} c_{\vartheta }c_{\psi } & c_{\vartheta }s_{\psi } & -s_{\vartheta }\\ s_{\varphi }s_{\vartheta }c_{\psi }-c_{\varphi }s_{\psi } & s_{\varphi }s_{\vartheta }s_{\psi }+c_{\varphi }c_{\psi } & s_{\varphi }c_{\vartheta }\\ c_{\varphi }s_{\vartheta }c_{\psi }+s_{\varphi }s_{\psi } & c_{\varphi }s_{\vartheta }s_{\psi }-s_{\varphi }c_{\psi } & c_{\varphi }c_{\vartheta } \end{array}]\;.$$

The gravity vector is therefore represented in the body-fixed coordinate system as follows:

(19) $$g_{B}=\left[\begin{array}{ccc} c_{\vartheta }c_{\psi } & c_{\vartheta }s_{\psi } & -s_{\vartheta }\\ s_{\varphi }s_{\vartheta }c_{\psi }-c_{\varphi }s_{\psi } & s_{\varphi }s_{\vartheta }s_{\psi }+c_{\varphi }c_{\psi } & s_{\varphi }c_{\vartheta }\\ c_{\varphi }s_{\vartheta }c_{\psi }+s_{\varphi }s_{\psi } & c_{\varphi }s_{\vartheta }s_{\psi }-s_{\varphi }c_{\psi } & c_{\varphi }c_{\vartheta } \end{array}\right]\;\left[\begin{array}{c} 0\\ 0\\ g \end{array}\right]\;=g[\begin{array}{c} -s_{\vartheta }\\ s_{\varphi }c_{\vartheta }\\ c_{\varphi }c_{\vartheta } \end{array}]\;.$$

Equation (19) shows that a body-fixed tri-axial accelerometer does not convey heading information.

The time rates of change of the Euler angles are related to the components of the angular velocity **ω**_B_ = [*p q r*]*^T^* resolved in the body-fixed frame by the following system of first-order nonlinear differential equations [31]:

(20) $$\left[\begin{array}{c} \frac{d\varphi }{\mathrm{dt}}\\ \frac{d\vartheta }{\mathrm{dt}}\\ \frac{d\psi }{\mathrm{dt}} \end{array}\right]=\left[\begin{array}{ccc} 1 & \frac{s_{\varphi }s_{\vartheta }}{c_{\vartheta }} & \frac{s_{\varphi }s_{\vartheta }}{c_{\vartheta }}\\ 0 & c_{\varphi } & -s_{\varphi }\\ 0 & \frac{s_{\varphi }}{c_{\vartheta }} & \frac{c_{\varphi }}{c_{\vartheta }} \end{array}\right]\;[\begin{array}{c} p\\ q\\ r \end{array}]\;.$$

Equation (20) can be used to update the orientation of the rigid body in time given the angular velocity. As for all unconstrained representations of orientation, Euler angles suffer from singularities, commonly referred to as gimbal-lock: for instance, in the case of the 3-2-1 rotation sequence, if the pitch angle ϑ is ± *π*/2, the last two terms of the first and last rows in (20) go to infinite and the Euler angle integration becomes indeterminate. Gimbal lock corresponds to loosing a degree of freedom in the rotation matrix (18); for instance, when ϑ is *π*/2, the rotation matrix becomes:

(21) $$R\left(\psi ,\frac{\pi }{2},\varphi \right)=[\begin{array}{ccc} 0 & 0 & 1\\ s_{\varphi -\psi } & c_{\varphi -\psi } & 0\\ c_{\varphi -\psi } & -s_{\varphi -\psi } & 0 \end{array}]\;.$$

The rotation depends on the difference *φ* –*ψ*; only one degree of freedom therefore exists instead of two. In other terms, changes of *φ* and *ψ* result in rotations about the same axis.

Finally, since matrix multiplication is not generally commutative, finite rotations in space do not commute, unless infinitesimal rotation angles δ*ψ*, δθ, δ*φ* are considered, in which case we have:

(22) $$R\left(\delta \psi ,\delta \vartheta ,\delta \varphi \right)\approx [\begin{array}{ccc} 1 & \delta \psi & -\delta \vartheta \\ -\delta \psi & 1 & \delta \varphi \\ \delta \vartheta & -\delta \varphi & 1 \end{array}]\;.$$

In fact, the Euler’s formula (15) can be approximated to first order as follows:

(23) $$R\left(n,\delta \theta \right)\approx I_{3}-\left[\delta \theta \times \right]+O\left({\left|\delta \theta \right|}^{2}\right)\approx [\begin{array}{ccc} 1 & \delta \theta_{3} & -\delta \theta_{2}\\ -\delta \theta_{3} & 1 & \delta \theta_{1}\\ \delta \theta_{2} & -\delta \theta_{1} & 1 \end{array}]\;.$$

where *δ***θ** = *δθ***n** is the infinitesimal rotation vector, and its components δ*θ*_1_, δ*θ*_2_, δ*θ*_3_ are termed infinitesimal angles.

Finally, another mathematical representation of orientation can be constructed by rewriting the Euler’s formula (15) as follows:

(24) $$R\left(q,q_{4}\right)=[\begin{array}{ccc} q_{1}^{2}-q_{2}^{2}-q_{3}^{2}+q_{4}^{2} & 2\left(q_{1}q_{2}+q_{3}q_{4}\right) & 2\left(q_{1}q_{3}-q_{2}q_{4}\right)\\ 2\left(q_{1}q_{2}-q_{3}q_{4}\right) & -q_{1}^{2}+q_{2}^{2}-q_{3}^{2}+q_{4}^{2} & 2\left(q_{2}q_{3}+q_{1}q_{4}\right)\\ 2\left(q_{1}q_{3}+q_{2}q_{4}\right) & 2\left(q_{2}q_{3}-q_{1}q_{4}\right) & -q_{1}^{2}-q_{2}^{2}+q_{3}^{2}+q_{4}^{2} \end{array}]\;.$$

The rotation matrix (24) is formulated as a homogeneous quadratic function of the quantities *q_i_*, *i* = 1,…,4, called the Euler-Rodrigues symmetric parameters or quaternion [32]:

(25) $$R\left(q,q_{4}\right)=\left(q_{4}-{\left|q\right|}^{2}\right)\;I_{3}+{2\mathrm{qq}}^{T}-2q_{4}\left[q\times \right],$$

where:

(26) $$q=\left[\begin{array}{c} q_{1}\\ q_{2}\\ q_{3} \end{array}\right]=\text{sin}\;\left(\frac{\theta }{2}\right)\;n,\;q_{4}=\text{cos}\;\left(\frac{\theta }{2}\right).$$

It is commonplace to refer to **q** as the vector part and to *q*_4_ as the scalar part of the quaternion **q̄** = [**q***^T^* *q*_4_]*^T^*. As implied by (26), the rotation quaternion satisfies the simple normalization constraint:

(27) $$q_{4}+{\left|q\right|}^{2}=1.$$

The following two basic operations are defined in the quaternion space:

(28) $$\begin{array}{l} \text{addition}\\ \bar{q}⊕{\bar{q}}^{\prime }={\left[{\left(q+q^{\prime }\right)}^{T}\;q_{4}+q_{4}^{\prime }\right]}^{T}\\ \text{multiplication}\\ \bar{q}⊗{\bar{q}}^{\prime }={\left[{\left(q_{4}^{\prime }q+q_{4}q^{\prime }+q\times q^{\prime }\right)}^{T}\;q_{4}\;q_{4}^{\prime }-q\cdot q^{\prime }\right]}^{T} \end{array}$$

In contrast with quaternion addition, quaternion multiplication is not generally commutative. Moreover, by analogy with complex numbers, we define the conjugate of a quaternion; the definitions of quaternion norm and inverse follow:

(29) $$\begin{array}{l} \text{conjugate}\\ {\bar{q}}^{*}={\left[-q^{T}\;q_{4}\right]}^{T}\;\to \;{\bar{q}}^{*}⊗\bar{q}={\left[0^{T}\;q_{4}+{\left|q\right|}^{2}\right]}^{T}\\ \text{norm}\\ \left|\bar{q}\right|=\sqrt{{\bar{q}}^{*}⊗\bar{q}}=q_{4}+{\left|q\right|}^{2}=\sum_{i=1}^{4}q_{i}^{2}\\ \text{inverse}\\ {\bar{q}}^{\;-1}\;\text{such that}\;{\bar{q}}^{-1}⊗\bar{q}={\left[0^{T}\;1\right]}^{T}\;\to \;{\bar{q}}^{\;-1}=\frac{{\bar{q}}^{*}}{{\left|\bar{q}\right|}^{2}} \end{array}$$

The Euler-Rodrigues formulation predates the discovery of quaternions by Hamilton, who was not apparently interested in developing quaternion algebra as means of describing rotational transformations [31]. Hamilton’s quaternions can be considered as 4-component extended complex numbers of the form:

(30) $$\bar{q}=q_{1}\;i+q_{2}\;j+q_{3}\;k+q_{4},$$

whose imaginary components **i**, **j**, **k** have the computation rules:

(31) i⊗i=j⊗j=k⊗k=i⊗j⊗k=−1, i⊗j=k, j⊗i=−k.

Alternatively, quaternions can be considered as vectors embedded in the four-dimensional Euclidean space R^4^. The set of quaternions with null vector parts can be identified with R the set of quaternions with null scalar part, aka vector quaternions, can be identified with vectors in the Euclidean space R^3^. At last, unit quaternions, namely quaternions with unit norm, lie on the three-dimensional sphere S^3^ with unit radius in R^4^. Henceforth, the vector presentation is used in place of the representation as extended complex numbers.

The connection existing between unit quaternions and the problem of describing orientations starts with examining (24–26). Given the vector quaternion **p̄** = [**p***^T^* 0]*^T^*, the vector quaternion:

(32) $${\bar{p}}^{\prime }={\bar{q}}^{\;-1}⊗\bar{p}⊗\bar{q}$$

is shown to be **p** rotated about the **n**-axis through an angle *θ* [32]. Any general three-dimensional rotation *θ* about an arbitrary unit vector **n** can be therefore described by a unit quaternion. The rule of composition of rotations is achieved by multiplying the corresponding quaternions. Let **q̄**_1_ and **q̄**_2_ be arbitrary unit quaternions. Rotation by **q̄**_1_ followed by rotation by **q̄**_2_ is shown equivalent to rotation by **q̄**_2_ ⊗ **q̄**_1_

(33) $$R\left(\bar{q}\right)=R\left({\bar{q}}_{2}\right)\;R\left({\bar{q}}_{1}\right)\;↔\;\bar{q}={\bar{q}}_{2}⊗{\bar{q}}_{1}$$

The four-component unit quaternion has the lowest dimension of any globally non-singular orientation parameterization. Enforcing the unit norm constraint on a quaternion leaves it with the three degrees of freedom consistent with the SO(3) dimensionality. Moreover, the quaternion representation is redundant, as the rotation vector. The quaternion −**q̄** represents the same rotation as **q̄** a rotation through the angle *θ* about the **n**-axis can also be expressed as a rotation through an angle −*θ* about the **n**’-axis (**n**’ = −**n**).

### 3.2. Kinematic Equations Describing the Motion of a Rigid Body

The kinematic equations that describe the motion of a rigid body capture the relations existing between the temporal derivative of the orientation representation and the angular velocity; we have already discussed the formulation of these equations in the case that the 3-2-1 rotation sequence of Euler’s angles are chosen for representing the orientation, see (20).

Suppose that the orientation changes with time: ${}_{E}^{B}C\;\left(t+\delta t\right)$, *i.e.*, the rotation matrix representing the orientation at time *t* + δ*t*, differs from ${}_{E}^{B}C\;\left(t\right)$, the rotation matrix at time *t*, see (23):

(34) $${}_{E}^{B}C\;\left(t+\delta t\right)=\Phi \;\left(t,t+\delta t\right)\;{}_{E}^{B}C\;\left(t\right)\;\leftarrow \;\Phi \;\left(t,t+\delta t\right)=I_{3}-\left[\delta \theta \;\left(t\right)\times \right]+O\left({\left|\delta \theta \right|}^{2}\right).$$

where *δ* **θ**(*t*) is the infinitesimal rotation vector. We can write:

(35) $$\frac{{}_{E}^{B}C\;\left(t+\delta t\right)-{}_{E}^{B}C\;\left(t\right)}{\delta t}=-\frac{\left[\delta \theta \;\left(t\right)\times \right]}{\delta t}{}_{E}^{B}C\;\left(t\right).$$

Taking the limit of (35) as *δ t* tends to zero, one obtains a system of first-order linear differential equations, aka the Poisson’s kinematic equations:

(36) $$\frac{d}{\mathrm{dt}}{}_{E}^{B}C=-\left[\omega_{B}\times \right]{}_{E}^{B}C.$$

where **ω***_B_* is the body-referenced angular velocity, defined as:

(37) $$\omega_{B}=\underset{\delta t\to 0}{\text{lim}}\frac{\delta \theta }{\delta t}.$$

The time dependence of the angular velocity and the rotation matrix is not made explicit in (36)–(37) to avoid unnecessary cluttering of the notation. Alternatively, the time evolution of a time-varying quaternion with angular velocity **ω***_B_* is given by the solution to the following system of first-order linear differential Equation [32]:

(38) $$\frac{d}{\mathrm{dt}}\bar{q}=\frac{1}{2}\bar{q}⊗{\left[\omega_{B}^{T}\;0\right]}^{T}=\frac{1}{2}\left[\begin{array}{cc} -\left[\omega_{B}\times \right] & \omega_{B}\\ -\omega_{B}^{T} & 0 \end{array}\right]\;\bar{q}=\Omega \left(\omega_{B}\right)\;\bar{q},$$

where **Ω**(**ω**_B_) is a 4 × 4 skew symmetric matrix. If the angular velocity is time constant, then the closed-form solution to (38) with given initial conditions is given by:

(39) $$\bar{q}\;\left(t\right)=\Phi \;\left(t,t_{0};\omega_{B}\right)\;\bar{q}\;\left(t_{0}\right)=\text{exp}\;[\left(t-t_{0}\right)\Omega \;\left(\omega_{B}\right)]\;\bar{q}\;\left(t_{0}\right).$$

The matrix exponential can be written:

(40) $$\Phi \left(t_{0},t;\omega_{B}\right)=\text{cos}\;(\left|\omega_{B}\right|\frac{t-t_{0}}{2})\;I_{4}\;+\;\frac{\text{sin}\;\left(\left|\omega_{B}\right|\frac{t-t_{0}}{2}\right)}{\left|\omega_{B}\right|\frac{t-t_{0}}{2}}\Omega \left(\omega_{B}\right).$$

It is worth noting that all three-dimensional representations of orientation are invariably associated to non-linear kinematic equations; on the other hand, higher-dimensional representations of orientation, such as the orientation matrix and the quaternion, present linear kinematic equations, as shown in (36–38).

The gimbal-lock singularity and the presence of computationally taxing trigonometric functions in the numerical integration of the system (20) are critical elements against the choice of the Euler angles. Mathematically, (36) preserves the orthogonality of the orientation matrix, although errors associated with its numerical integration can cause some degradation in the orthogonality of the matrix, which forces to adopt suitable methods to recover it [33]. Errors associated with numerical integration of the kinematic equations for orientation have been analyzed and characterized for both the rotation matrix and the quaternion parameterizations, and the superiority of the latter is widely recognized [33,34]. In addition to that, another relevant advantage of the quaternion formulation is vastly increased computational speed: trigonometric functions are not to be computed, with further savings that are provided by the reduced number of floating operations involved in numerically integrating (38) as compared with (36) [31].

The claim that the physical interpretation of the quaternion is much less intuitive than that associated with Euler angles does not imply that the quaternion cannot find ample diffusion even in the biomechanical community. A systematic development of the kinematics equations is possible in terms, equivalently, of direction cosines, the rotation vector, Euler angles, the quaternion, and well-established relationships link all these descriptors to one another: at any stage of the processing and visualization tasks, one can adopt the descriptor that is more suited to the application specifics. Henceforth, we direct our attention exclusively to the quaternion-based formulation of the kinematic equations of a rigid body.

## 4. Orientation Estimation Algorithms

Estimating the orientation from body-fixed sensor measurements has a quite long history, in particular in applications of spacecraft guidance and control. Over the years, two main approaches have emerged: the deterministic (least-squares) approach and the stochastic (Kalman filtering) approach. The least-squares approach was originally introduced in 1965, in the so-called Wahba’s problem [35], which is a constrained least-squares optimization problem for finding the rotation matrix from vector measurements taken at a single time (single-frame method). The Kalman filtering approach, first proposed in 1961 for applications of spacecraft guidance and control [36], soon after the publication of the seminal paper by Kalman in 1960 [37], is intended to yield minimum-variance sequential estimates of orientation and, in principle, of other parameters than orientation, such as sensor biases, using information about motion dynamics. Unless suitable generalizations are provided, deterministic approaches are unable to incorporate such information [38,39].

Estimating the orientation of human body parts from body-fixed inertial/magnetic sensor measurements is a relatively novel application. It does not come as a surprise that the same distinction as above is made between deterministic and stochastic approaches, as we will see shortly after.

### 4.1. Deterministic Single-Frame Approach

Deterministic single-frame estimation algorithms can be proposed in connection with the operation of gyro-free aiding sensor systems. Four variants of the same approach are surveyed here: TRIAD (TRi-axial Attitude Determination), QUEST (QUaternion ESTimator), FQA (Factored Quaternion Algorithm) and Gauss-Newton (GN) optimization. They can be used to solve Wahba’s problem without the need for an *a priori* estimate. They all are based on the concept of vector matching, which requires, in principle, that measurements of constant reference vectors (e.g., gravity and earth magnetic field) are performed. In their original formulation, they are unable to provide sequential estimates of a time-varying orientation and of other parameters than the orientation, such as sensor biases. In the presence of uncompensated sensor biases, the estimated orientation can be therefore grossly inaccurate.

Suppose that two nonparallel reference unit vectors **v**_1_, **v**_2_ are available, e.g., in the direction of the gravity field and the earth magnetic field, and resolved in the earth-fixed frame. The corresponding observation vectors **w**_1_, **w**_2_ are measured in the body-fixed frame and normalized in amplitude to one. The TRIAD algorithm attempts to solve Wahba’s problem by finding an orthogonal matrix **A** such that the pair (**w**_1_, **w**_2_) is optimally related to the pair (**v**_1_, **v**_2_), namely **Av***_i_* = **w***_i_*, *i* = 1, 2, which gives rise to an over-determined system of algebraic equations [40].

First, two triads of orthonormal reference and observation vectors are constructed:

(41) $$\begin{array}{l} r_{1}=v_{1};\;r_{2}=\frac{r_{1}\times v_{2}}{\left|r_{1}\times v_{2}\right|};\;r_{3}=r_{1}\times r_{2}\\ s_{1}=w_{1};\;s_{2}=\frac{s_{1}\times w_{2}}{\left|s_{1}\times w_{2}\right|};\;s_{3}=s_{1}\times s_{2}. \end{array}$$

Second, the two orthogonal matrices **M**_ref_ and **M**_obs_ are formed, and the optimal estimate of the orthogonal matrix **A** is then computed as follows:

(42) $$\{\begin{array}{l} M_{\text{ref}}=\left[r_{1}\;r_{2}\;r_{3}\right]\\ M_{\text{obs}}=\left[s_{1}\;s_{2}\;s_{3}\right] \end{array}\;\to \;A=M_{\text{obs}}M_{\text{ref}}^{T}.$$

The main disadvantage of the TRIAD algorithm is that it is sensitive to the order at which the algorithm receives the two vector pairs—the pair (**v**_1_, **w**_1_) is received first in (41). In fact, part of the information conveyed by the second vector pair is discarded: the cross products that are needed to compute **r**_2_ and **s**_2_ eliminate any contribution of **v**_2_ and **w**_2_ relative to the vertical axis. Since the accuracy of the orientation estimate is more influenced by the vector pair that is processed first, the best choice would be to process first the observation vector of greater accuracy. Another disadvantage of the TRIAD algorithm is that it accommodates only two observation vectors.

The basic QUEST delivers the optimal quaternion that minimizes the loss function:

(43) $$L\left(A\right)=\frac{1}{2}\sum_{i=1}^{n}a_{i}{\left|w_{i}-{\mathrm{Av}}_{i}\right|}^{2}.$$

The loss function (44) can be transformed into a quadratic gain function of the unit quaternion:

(44) $$G\left(A\left(\bar{q}\right)\right)={\bar{q}}^{T}K\;\bar{q}$$

where **K** is a 4 × 4 matrix constructed from the reference vectors **v***_i_*, measurement vectors **w***_i_*, and weighting coefficients *a_i_*. The optimal unit quaternion is proven to be the eigenvector of the **K** matrix corresponding to its largest eigenvalue [40].

In contrast with the TRIAD algorithm, the QUEST is capable of accommodating more than two observation vectors; moreover, it is optimal with sensors with different accuracies by properly selecting the weighting coefficients *a_i_*. Although the quaternion produced by the QUEST is unit-norm and globally non-singular, a method is needed for avoiding the singularity that arises when the angle of rotation is *π*. In fact, the QUEST uses a three-dimensional parameterization, namely the Gibbs vector, in its derivation:

(45) $$a_{g}=\frac{\bar{q}}{q_{4}}=n\;\text{tan}\frac{\theta }{2}$$

The singularity problem is eliminated in the QUEST by employing the method of sequential rotations, at the expense of computational cost [40].

The FQA is specifically created in the attempt to overcome the limitation of both the TRIAD algorithm and the QUEST that orientation errors arise from errors in just one of the sensor data [41]. Suppose that the two reference vectors are the gravity field, **g**, normalized in amplitude to one (vertical reference); and the earth's magnetic field, or more precisely, the local magnetic field, **h**, normalized in amplitude to one (horizontal reference). Let **g**_m_ and **h**_m_ denote the corresponding measurement vectors, normalized in amplitude to one. In the FQA, acceleration data are used in computing the pitch and roll angles, while local magnetic field data are used only in yaw angle computations. This decoupling eliminates the influence of magnetic variations on calculations that determine pitch and roll angles.

Upon examination of (19), the value of the sine of the pitch angle can be expressed as:

(46) $$\text{sin}\;\vartheta =g_{\mathrm{mx}}\;\to \;\text{cos}\;\vartheta =\sqrt{1-{\text{sin}}^{2}\;\vartheta },\;\vartheta \in ]-\pi /2,\;\pi /2].$$

From trigonometric half-angle formulas, half-angle values are given by:

(47) $$\begin{array}{l} \text{sin}\frac{\vartheta }{2}=\text{sign}\;\left(\text{sin}\;\vartheta \right)\sqrt{\left(1-\text{cos}\;\vartheta \right)/2}\\ \text{cos}\;\frac{\vartheta }{2}=\sqrt{\left(1+\text{cos}\;\vartheta \right)/2}. \end{array}$$

yielding the following expression of the pitch quaternion:

(48) $${\bar{q}}_{p}={\left[0\;\text{sin}\frac{\vartheta }{2}\;0\;\text{cos}\frac{\vartheta }{2}\right]}^{T}$$

The values of the sine and cosine of the roll angle can be expressed as:

(49) $$\begin{array}{l} \text{sin}\;\varphi =-g_{\mathrm{my}}/\text{cos}\;\vartheta \\ \text{cos}\;\varphi =-g_{\mathrm{mz}}/\text{cos}\;\vartheta . \end{array}$$

Exploiting the half-angle formulas, the roll quaternion is given by:

(50) $${\bar{q}}_{r}={\left[\text{sin}\frac{\varphi }{2}\;0\;0\;\text{cos}\frac{\varphi }{2}\right]}^{T}$$

The values of the sine and cosine of the yaw angle can be determined by matching the magnetic field reference vector in the horizontal plane [*h_x_* *h_y_*]*^T^*, normalized in amplitude to one, and the measured magnetic vector [*m_x_* *m_y_*]*^T^*, projected from the body-fixed frame to the horizontal plane via the pitch and roll quaternions, and normalized in amplitude to one:

(51) $$\left[\begin{array}{c} \text{cos}\;\psi \\ \text{sin}\;\psi \end{array}\right]=\left[\begin{array}{cc} m_{x} & m_{y}\\ -m_{y} & m_{x} \end{array}\right]\;[\begin{array}{c} h_{x}\\ h_{y} \end{array}].$$

Exploiting the half-angle formulas, the yaw quaternion is:

(52) $${\bar{q}}_{y}={\left[0\;0\;\text{sin}\frac{\psi }{2}\;\text{cos}\frac{\psi }{2}\right]}^{T}$$

The rule of composition (33) is applied to (48), (50) and (52) to yield the quaternion estimate representing the orientation of the rigid body:

(53) $$\bar{q}={\bar{q}}_{r}⊗{\bar{q}}_{p}⊗{\bar{q}}_{y}.$$

**Figure 4.**
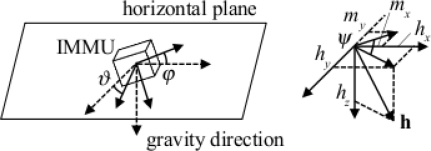
Inclination of the strap-down magnetic compass relative to the horizontal plane as defined by gravity direction.

Since the FQA uses three angles to derive the quaternion estimate, it suffers from a singularity, which occurs when the pitch angle is ± π/2. In order to circumvent this singularity, a method similar to the one proposed in the QUEST algorithm is adopted in the numerical implementation of the FQA. In essence, the FQA is very similar to the tilt compensation procedure customarily used in a strap-down magnetic compass to derive heading [17], Figure 4.

The last method reviewed in this Section is the GN optimizer [42]. First, construct the error vector as follows:

(54) $$ɛ\left(\bar{q}\right)=\left[\begin{array}{c} g_{m}-A\left(\bar{q}\right)\;g\\ \rho \left(h_{m}-A\left(\bar{q}\right)\;h\right) \end{array}\right]$$

where *ρ* is a suitably chosen weighting factor. Then, the optimal quaternion is computed by minimization of the square of the error vector:

(55) $${\bar{q}}_{\text{opt}}=\underset{\bar{q}}{\text{arg min}}\left\{\frac{1}{2}ɛ{\left(\bar{q}\right)}^{\;T}ɛ\left(\bar{q}\right)\right\}$$

The conventional GN method algorithm provides an iterative solution to this problem:

1. Give an initial guess, **q̄**_0_;
2. Compute the correction:
(56) $$\Delta \bar{q}={\left({\left(\frac{\partial \;ɛ}{\partial \;\bar{q}}\right)}^{T}\left(\frac{\partial \;ɛ}{\partial \;\bar{q}}\right)\right)}^{-1}{\left(\frac{\partial \;ɛ}{\partial \;\bar{q}}\right)}^{T}ɛ\left(\bar{q}\right);$$
3. Compute the updated quaternion:
(57) $${\bar{q}}_{i+1}={\bar{q}}_{i}-\Delta \bar{q};$$
4. Normalize the updated quaternion:
(58) $${\bar{q}}_{i+1}=\frac{{\bar{q}}_{i+1}}{\left|{\bar{q}}_{i+1}\right|};$$
5. Return to step (2), and repeat until convergence using the stopping criterion:
(59) $$\left|\Delta \bar{q}\right|<\text{TOL},$$

where TOL measures how small the residual of the final solution is to be considered acceptable.

The main merit of a GN optimizer is recognized in its robust estimation capability, nonetheless the intensive calculations required in step (2) and the need for iteratively evaluations until convergence may diminish its importance for real-time applications. It is reported that four-five iterations are requested for typical values of the sensor measurement noise, when the initialization errors are large. Fortunately, during tracking, when errors are much smaller, GN iteration typically converges to sufficient accuracy in only one-two steps. Clever algorithms are reported in the literature in order to reduce the computational burden of GN optimizers [25,42].

For an approach based on single-frame deterministic algorithms to work properly in human motion tracking, the acceleration and magnetic measurement vectors are to be determined by the gravity field and by the reference magnetic field, respectively. However, this assumption can cause serious errors in the orientation solution if any body accelerations and magnetic variations of affect the sensor signals: in principle, only slow motions occurring in magnetically clean environments would be allowed. The widespread practice of low-pass filtering acceleration signals in order to reduce the effect of dynamic motions leads to latency in the estimates produced by the algorithm, with the additional problem of how to optimally select the cut-off frequency of the filter. Alternatively, acceleration measurements would be screened before their use in the algorithm by computing the absolute value of the difference between their norm and the known value of gravity; if the computed value exceeds a preset threshold value, the measurement reliability is considered low. As for the impact of magnetic variations on the reliability of magnetic measurements, a similar approach can be considered by comparing the norm of the sensed magnetic field with the norm of the reference magnetic vector. A more detailed explanation of these vector selection techniques is deferred to Section 5.4, after that, in the next Section, stochastic estimation algorithms are presented and discussed.

### 4.2. Stochastic Estimation Algorithms

Stochastic estimation algorithms use a model for predicting aspects of the time behaviour of a system (dynamic model) and a model of the sensor measurements (measurement model), in order to produce the most accurate estimate possible of the system state. KF algorithms lend themselves perfectly to this task [37]. There appears to be wide consensus that, e.g., in the VR/AR community the KF is recognized “perhaps *the* perfect tool for elegantly combining multisensory fusion, filtering, and motion prediction in a single fast and accurate framework” [43].

For the sake of generality, our discussion starts here with considering the Bayesian approach to dynamic state estimation [44]; KFs represent a special class of algorithms for recursive Bayesian state estimation. The Bayesian approach is based on propagating the probability density function (PDF) of the system state in a recursive manner through the application of the Bayes’ rule. The state dynamics is modelled as a Markov process:

(60) $$p\;\left(x_{k}|x_{1:k-1}\right)=p\;\left(x_{k}|x_{k-1}\right),$$

where x_1:*k*−1_ = [**x**_1_ **x**_2_ … **x**_*k*−1_] is the collection of the states traversed by the system up to time *k*−1, included. The state at time *t_k_* is conditioned only on the previous state and it is independent of the past. This allows for a state representation according to the following discrete-time stochastic model:

(61) $$x_{k}=f_{k-1}\;\left(x_{k-1},w_{k-1}\right),$$

where *f* is a (linear or non-linear), generally time-variant function mapping the previous state to the current state, and **w**_*k*−1_ represents the process noise. Process noise accounts for any mismodelling effects or disturbances in the dynamic model. Here the index *k* is associated to a continuous-time instant *t_k_*, and the sampling interval *T*_*k*−1_ = *t_k_* – *t*_*k*−1_ may be time-dependent, *i.e.*, function of *k*. Henceforth, we will assume that the sampling interval *T_s_* is constant.

The system state **x***_k_* is related to the measurements by the measurement model:

(62) $$z_{k}=h_{k}\;\left(x_{k},v_{k}\right),$$

where **z***_k_* is the measured state of the process at time *t_k_*, *h* is a generally nonlinear time-variant function mapping of the state of the system to the measured state **z***_k_*, and **v***_k_* represents the measurement noise. The random processes **w**_*k*−1_ and **v***_k_* are assumed to be white, with known PDFs, and mutually independent. The initial state **x**_0_ is assumed to have a known PDF *p*(**x**_0_) and also to be independent of **w**_*k*−1_ and **v***_k_*.

The goal of filtering can be stated as finding estimates of the states given **z**_1:*k*_. This requires the calculation of the posterior PDF *p*(**x**_*k*_|**z**_1:*k*_). Suppose that the required posterior PDF *p*(**x**_*k*−1_|**z**_1:*k*−1_) at time *t*_*k*−1_ is available. The prediction stage involves the dynamic model (61) to obtain the prior PDF of the state at time *t_k_*:

(63) $$p\;\left(x_{k}|z_{1:k-1}\right)=\int p\;\left(x_{k}|x_{k-1}\right)\;p\;\left(x_{k-1}|z_{1:k-1}\right)\;{dx}_{k-1}.$$

The transitional PDF *p*(**x***_k_*|**x**_*k*−1_) is defined by the dynamic model (62) and the known statistics of the process noise **w**_*k*−1_.

The update stage, at time *t_k_* when a new measurement becomes available, is based on the Bayes’ rule:

(64) $$p\left(x_{k}|z_{1:k}\right)\propto p\;\left(z_{k}|x_{k}\right)\;p\;\left(x_{k}|z_{1:k-1}\right),$$

where the likelihood function *p*(**z***_k_*|**x***_k_*) is defined by the measurement model (62) and the known statistics of the measurement noise **v***_k_*.

The Equations (63)–(64) give a recursive way to propagate the posterior density. Bayesian filtering can thus be seen as a two-stage process, a prediction stage of the new state using (63), and an update stage where the prediction is modified by the new measurement using (64). The knowledge of the posterior PDF *p*(**x***_k_*|**z**_1:*k*_) allows estimating the state, and obtaining measures of the accuracy of these estimates. The Bayesian solution cannot be determined analytically, expect that in a restrictive set of cases, including the KF.

The KF assumes that the posterior density at every time step is multivariate Gaussian, and hence it can be completely characterized by the mean vector and the covariance matrix. If the PDF *p*(**x**_*k*−1_|**z**_1:*k*−1_) is Gaussian, the PDF *p*(**x***_k_*|**z**_1:*k*_) is proven to be Gaussian provided that:

- **w**_*k*−1_ and **v***_k_* are drawn from Gaussian PDFs with known parameters;
- *f*_*k*−1_(**x**_*k*−1_, **w**_*k*−1_) is a known *linear* function of **x**_*k*−1_ and **w**_*k*−1_;
- *h_k_*(**x***_k_*, **v***_k_*) is a known *linear* function of **x***_k_* and **v***_k_*.

In other words (61)–(62) can be rewritten as:

(65) $$\begin{array}{l} x_{k}=F_{k-1}x_{k-1}+w_{k-1}\\ z_{k}=H_{k}x_{k}+v_{k}, \end{array}$$

where **F**_*k*−1_ (of dimension *n*_x_ × *n*_x_) and **H***_k_* (of dimension *n*_z_ × *n*_z_) are known matrices. The additive noises **w**_*k*−1_ and **v***_k_* are mutually independent zero-mean white Gaussian, with known covariance matrices **Q**_*k*−1_ and **R***_k_*, respectively. Note that the system and measurement matrices **F**_*k*−1_ and **H***_k_*, as well as the covariance matrices **Q**_*k*−1_ and **R***_k_*, are allowed to be time-variant. For the reader’s convenience, the KF equations are reported in Appendix A.

The Extended Kalman Filter (EKF) is derived for nonlinear systems with additive noise:

(66) $$\begin{array}{l} x_{k}=f_{k-1}\left(x_{k-1}\right)+w_{k-1}\\ z_{k}=h_{k}\left(x_{k}\right)+v_{k}. \end{array}$$

The additive noises **w**_*k*−1_ and **v***_k_* are mutually independent, zero-mean white Gaussian with known covariance matrices **Q**_*k*−1_ and **R***_k_*, respectively. The EKF is based on the assumption that local descriptions of the nonlinear functions *f*_*k*−1_(**x**_*k*−1_) and *h_k_*(**x***_k_*) can be obtained by approximating them using only the first term in the Taylor series expansion. The posterior PDF *p*(**x***_k_*|**z**_1:*k*_) is therefore approximated by a Gaussian density. The local linearization requires the computation of the Jacobian matrices of the dynamic model, the measurement model or both with current predicted states, see Appendix A.

The EKF and its many variants are referred to as analytic approximations because the Jacobian matrices **F**_*k*−1_ and **H***_k_* have to be computed analytically. Moreover, it is worthy noting that the EKF always approximates the posterior PDF *p*(**x***_k_*|**z**_1:*k*_) as a multivariate Gaussian. If the nonlinearity in models is severe, the non-Gaussian nature of the posterior PDF *p*(**x***_k_*|**z**_1:*k*_) can be pronounced, e.g., it can be multimodal, heavily-tailed or skewed: the approximation to first-order is grossly inaccurate and the performance of the EKF can therefore be seriously degraded. In general, besides the computational costs incurred in the calculations of the Jacobian matrices, other disadvantages of the linearization procedure implemented in an EKF concern the sensitivity to initial conditions, biases in the estimation errors, critical problems of convergence and filter stability, especially when the sampling interval is too small.

The Unscented Kalman Filter (UKF) is developed with the aim to overcome these limitations [45]. The UKF hinges on the assumption that it is easier to approximate a Gaussian distribution than it is to approximate an arbitrary nonlinear function. Instead of linearizing using Jacobian matrices, the UKF adopts a deterministic sampling approach to capture the estimates of the mean vector and the covariance matrix with a minimal set of sample points; this “capture” is accurate to the second-order Taylor series expansion for any nonlinearity. The UKF can be used with non-differentiable functions, it does not require the derivation of Jacobian matrices (derivative-free), and is valid to higher-order expansions than the standard EKF. Some work concerning applications for aircraft guidance and control shows the superiority of UKF over EKF, particularly in the presence of large initialization errors [46]. However, this is not usually the case with human body motion tracking applications. This is because, mostly, the filter operation starts with the human body being typically at rest within magnetically clean regions, and well-calibrated inertial/magnetic sensors, see Section 5. Hence, a gyro-free aiding sensor system can feed the EKF with accurate data for initialization at first contact. Moreover, there appears to be wide consensus that, in human body motion capture applications, although the EKF and the UKF may have roughly the same accuracy, the computational overhead of the UKF, the simplicity of the calculations of the Jacobian matrices, and the quasi-Gaussian nature of the posterior PDF *p*(**x***_k_*|**z**_1:*k*_) contribute to make the EKF a preferred choice, even in the most demanding scenarios [47,48]. In order to keep the length of this paper within reasonable limits, UKF and more advanced Bayesian filters, namely particle filters [48,49], are not further addressed here.

## 5. Designing a Quaternion-Based EKF for Orientation Determination

In applying Kalman filtering to the problem of inertial orientation tracking there is considerable freedom in dynamic and measurement modelling [50]. In this Section the discussion concerns how EKFs can be designed when the quaternion is chosen to represent the orientation. The main difficulty of using quaternion-based state vector components is in the application of the filter equations. This difficulty is due to the lack of independence of the four components of a quaternion, which are related by the constraint that the quaternion must have unit norm in order to represent a valid orientation. Constraints imposed on the estimated state variables cannot be preserved by EKFs in their standard development [51].

### 5.1. Dynamic Modelling

The most principled way to preserve the unit-norm property of the estimated quaternion is to create an algorithm where the error between the true and estimated quaternions is itself a quaternion and is multiplied (in the sense of quaternion multiplication) with the *a priori* quaternion estimate to yield the *a posteriori* estimate [51]. This kind of EKF is called multiplicative EKF (MEKF), which differs from the classic additive EKF (AEKF), which employs quaternion subtraction in place of quaternion multiplication [52]. An MEKF parameterizes the global orientation with a non-singular unit quaternion, while any unconstrained three-dimensional representation is used to represent the orientation errors [53]. The dynamic model in an MEKF describes therefore the kinematic equations of a rigid body in terms of the relationships existing between the three-dimensional orientation error and the angular velocity [46]. Strong similarities exist between the MEKF approach and the so-called indirect-state formulation of the Kalman process. In analogy with an MEKF, the indirect-state EKF includes a three-dimensional orientation error in the state vector [54]. The potential advantages of an indirect-state filter are that the state dimension is smaller as compared with a direct-state filter, with subsequent computational savings [50].

In spite that MEKFs are theoretically correct in treating the normalization constraint, most reported implementations regard the application of AEKFs. AEKFs relax the quaternion unit-norm constraint and treat the four components of the quaternion as independent parameters. A method to preserve the quaternion unit-norm property is to derive a sort of quaternion measurement model from the non-linear equation that expresses the unit-norm property (pseudo-measurement model) [47]. Another popular means is to normalize the *a posteriori* estimate after the measurement update stage (“brute-force” approach). Even though it is neither elegant nor optimal, the “brute-force” approach is often the preferred choice and is proven to work well [52]. The exemplary EKF developed in the following is direct-state, additive and enforces the unit-norm constraint by the “brute-force” approach.

The dynamic equations for describing the orientation of the parts to be tracked would cause severe difficulties in the filter modelling [55], and especially in human body motion capture applications, where the inputs, *i.e.*, muscle forces and torques, are unknown inputs [14,42]. The use of gyros as the primary means to estimate orientation allows circumventing these problems. Since the angular velocity of human body parts is obtained from the gyro data, the kinematic equations of a rigid body can be used to obtain the orientation state (model replacement). In other words, it is highly convenient to treat gyro data as external inputs to the filter rather than as measurements, and consequently gyro measurement noise and bias enter the filter as process noise rather than as measurement noise [22,56]. Another advantage of this choice is the reduction in the dimension of the state vector, which may lead to minimal-order, computationally efficient filter implementations.

An additional important feature of stochastic estimation algorithms like the EKFs is that gyro drift bias can be estimated by state vector augmentation techniques [57]. Oftentimes, this feature is exploited, especially for applications of air and spacecraft attitude estimation, so as to compensate the gyros before performing the numerical integration of kinematics equations [36,46,56]. This is very important in the case that the only aiding comes from occasional orientation fixes. Drift biases of the gyro-free aiding sensor system may be also estimated in the same way [14,22]. In general, however, these other biases cannot be estimated simultaneously with the orientation and gyro drift bias due to possible problems of system observability [58].

Most studies in VR/AR fields employ head motion trackers that directly provide orientation measurements. In these cases, quaternions can be used with an EKF to estimate the angular velocity, which is needed to predict the future head orientation. Sometimes, angular velocity is measured with inertial sensors; the state vector can therefore be augmented with additional components, *i.e.*, the angular acceleration [48]. Different motion models are implemented in the filtering algorithm, in order to improve the ability of the EKF to predict the head orientation for latency compensation [54,59]. For instance, the Constant Velocity (CV) model assumes a simple first-order Gauss-Markov (GM) model for each angular velocity component [60]:

(67) $$\frac{d\omega }{\mathrm{dt}}=-\frac{1}{\tau }\omega +\sqrt{\frac{{2\sigma }^{2}}{\tau }}w,$$

where *w* is a Gaussian white noise, with null mean and unit variance, *τ* is the decorrelation time constant of the GM model and *σ*^2^ is a variance factor. The GM model reflects underlying assumptions about the nature of human movements, namely, (a) the change of viewing direction is infrequent; (b) the angular velocity and acceleration are nonzero only during the infrequent changes in orientation. The decorrelation time constant and the variance factor are tuned in order that the spectral properties of the signal generated by the model match those of the angular velocities for paradigmatic motions [42]. More sophisticated models are also investigated, which opens the way to an interesting avenue of research concerning the development of multiple models of human (head) motion, and multiple model adaptive estimation techniques (MMAE) [61].

In this paper, the application specifics concern orientation measurement devices that use angular velocity to estimate orientation using an EKF. At the sampling intervals that are common for these devices, say between 100–500 Hz, the angular velocity can be considered constant in the time interval between successive measurements, leading to the numerical integration of kinematic equations via (39)–(40); the addition of some process noise may further help improving filter stability.

### 5.2. Sensor Modelling

In a fully integrated IMMU, the gyro, the accelerometer and the magnetometer are each tri-axial, with mutually orthogonal sensitivity axes. Their output in response to the body angular velocity **ω**_body_, acceleration (gravity **g** and body acceleration **a**_body_), and local magnetic field (earth’s magnetic field **h**_earth_ and some local magnetic effect modelled as a time-invariant magnetic vector **h**_ext_) are:

(68) $$\{\begin{array}{l} \omega_{B}={}^{g}K\;\omega_{\text{body}}+{}^{g}b+{}^{g}v\\ a_{B}={}^{a}K{}_{E}^{B}C\;\left(-g+a_{\text{body}}\right)+{}^{a}b+{}^{a}v\;\\ h_{B}={}^{h}K{}_{E}^{B}C\;\left(h_{\text{earth}}+h_{\text{ext}}\right)+{}^{h}b+{}^{h}v, \end{array}$$

where *^g^***K**, *^a^***K** and *^m^***K** are the matrices of the scale factors (ideally, they are equal to **I**_3_); *^g^***b**, *^a^***b** and *^h^***b** are the bias vectors (ideally, they are null); *^g^***v**, *^a^***v** and *^h^***v** are assumed uncorrelated white Gaussian measurement noise, with null mean and covariance matrix $\Sigma_{g}=\sigma_{g}^{2}I_{3}$, $\Sigma_{a}=\sigma_{a}^{2}I_{3}$ and $\Sigma_{h}=\sigma_{h}^{2}I_{3}$. Equation (68) is a simplified model that does not account for additional error sources, such as cross-axis sensitivity, gyro *g*-sensitivity, nonlinearity, hysteresis and misalignment [62]. It is worthy noting that a further simplification is made in the gyro model by omitting the earth’s angular velocity of 15°/hour, since state-of-the-art MEMS gyros are unable to sense this component. Their bias stability is indeed in the order of 1°/s—the bias stability is usually specified as a 1σ value, and it describes how the bias may change over a specified period of time, typically around 100 s, in fixed conditions (usually including constant temperature) [26].

**Figure 5.**
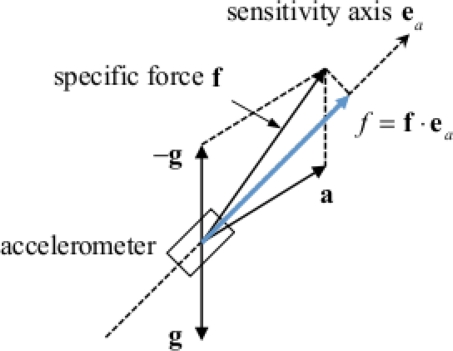
A single-axis accelerometer measures the projection (in the direction of the sensitive axis) of the specific force **f** resulting from the sum of the inertial acceleration **a** and the equivalent gravity acceleration −**g**.

To proceed in the discussion of the sensor model (68), remind that an accelerometer measures the projection along its sensitive axis of the specific force *f* it is submitted. The specific force additively combines the linear acceleration component *a*, due to body motion, and the gravitational acceleration component, –*g*, both projected along the sensitive axis of the accelerometer, Figure 5. In common parlance, the high-frequency component, aka the AC component, is related to the dynamic motion the subject is performing, e.g., walking, hand weaving, head shaking, and so forth, while the low-frequency component of the acceleration signal, aka the zero-frequency (DC) component, is related to the influence of gravity, and it can be exploited to identify static postures [63].

The bias and scale factor of inertial and magnetic sensors are functions of environmental conditions, in particular ambient temperature; this is especially true for gyros [64]. Temperature effects on accelerometers are of relatively lower quantitative relevance, and they are usually negligible on magnetic sensors across the thermal variations that they may encounter in practice. Moreover, scale factor drifts of inertial and magnetic sensors usually affect the accuracy of the measurement process to a much lesser extent than the bias drifts of these sensors. The influence of temperature on the gyro bias drift is particularly significant after that power is applied to gyros, as a result of device self-heating. Provided that gyros are allowed warm-up and thermal stabilization for few minutes, then their biases tend to change quite slowly with time. In practice, gyro bias errors can be calibrated and compensated effectively by so-called “zero attitude updates”, which require keeping the gyros from rotating. It is dependent on the nature of the specific application whether occasional rests can be assumed for the human body part to be monitored and tracked [65]. As for the scale factor calibration, procedures suited for in-field use are available [62,66]. The scale factor and bias errors of accelerometers can be calibrated and compensated by so-called “zero-velocity updates”, which require keeping the accelerometers from moving, although they are difficult to implement and may require specific manoeuvres to work properly [67]. In analogy with accelerometers, the scale factor and electronic bias errors of magnetic sensors can be calibrated and compensated effectively by in-field procedures [68]. In the case of experimental sessions lasting few minutes, it is quite safe to assume that the scale factor and bias errors of inertial and magnetic sensors are null, provided that the sensors are carefully calibrated before starting as explained above.

The problem of magnetic variations due to ferromagnetic materials in the vicinity of a magnetic sensor raises additional modelling considerations. Equation (68) shows that the reference magnetic vector is not necessarily the earth’s magnetic field **h**_earth_. The presence of any constant field vector **h**_ext_ superimposed on **h**_earth_ does not preclude the possibility of constructing the horizontal reference needed for orientation estimation, provided that **h**_ext_ is accurately known. This is in contrast with the need to perform ambulatory measurements, without prior knowledge of existence and location of disturbances. Moreover, we have to consider dynamic effects that are related to either ferromagnetic objects moving in the vicinity of the sensor or to movements of the body-fixed sensor relative to static ferromagnetic objects. Because of these dynamic effects, **h**_ext_ turns out to be time-variant and unknown. A strategy to tackle this problem is to assume that a time-variant bias error *^h^***b** is present in the magnetic sensor output. In the dynamic model the kinematics equations are therefore augmented with additional equations yielding the model of the behaviour in time of **h**_ext_ by “absorbing” it into a mathematical model of the magnetic drift bias, e.g., random-walk or first-order Gauss-Markov models (state augmentation). In other terms, the horizontal reference is built and maintained by the EKF itself (auto-calibration) [21,22].

### 5.3. Measurement Equations

The role of aiding sensors is played by accelerometers, taken alone or in combination with magnetic sensors. Sometimes, and depending on the requirements of a specific application, the complexity of the measurement hardware setup, and the sophistication of the filtering algorithms as well, can be reduced to some extent. Simplifying assumptions are quite common in applications to gait analysis [65]. For instance, in the case that motion outside the sagittal plane is assumed not to take place, accelerometers can be used without magnetic sensors, and, since the acceleration sensitivity axes can be embedded in the sagittal plane, simpler bi-axial configurations may suffice. Analogously, uni-axial gyros are enough to capture angular velocities when rotations are approximately about a single axis, oriented in the medio-lateral direction (orthogonal to the sagittal plane).

If motion occurs in the 3D space, heading estimation requires a horizontal reference, for which construction an IMMU is necessary. Few possibilities exist as for the choice of the measurement model. Since we prefer to take the measured angular velocity as an input to the filter, we have to decide how to handle acceleration and magnetic measurements. They can be fused together directly using any deterministic attitude estimation algorithm, e.g., TRIAD, QUEST, FQA, GN optimizer. An advantage of single-frame deterministic algorithms that deliver the quaternion at their output is that the measurement equations are linear; with the exception of the GN optimizer, see (57)–(58) in this regard, the unit-norm property of the measured quaternion is also preserved. The FQA is a potentially good choice, because of the decoupling of magnetic and acceleration data in estimating heading and inclination. A difficulty with this approach is in constructing the expression of the measurement noise covariance, a problem apparently dismissed in [41]. Analytical expressions of the covariance matrix for the TRIAD algorithm and QUEST are derived in [40]: it is proven that the noise in the quaternion measurements presents quaternion-dependent covariance matrices. The dependence on the quaternion is not a problem *per se*, since the predicted state can be used in place of the true unknown state. An element of complication is that the noisy reference magnetic vector estimated by the EKF must be used in the process of vector matching.

Alternatively, each reference vector component is given a specific measurement equation, which helps moving its representation from the earth-fixed to the body-fixed frame via either (24) or the rule of composition (33):

(69) $$\left[\begin{array}{c} a_{k}\\ h_{k} \end{array}\right]=\left[\begin{array}{cc} {}_{E}^{B}C\;\left({\bar{q}}_{k}\right) & 0\\ 0 & {}_{E}^{B}C\;\left({\bar{q}}_{k}\right) \end{array}\right]\;\left[\begin{array}{c} g\\ h \end{array}\right]+\left[\begin{array}{c} {}^{a}v{}_{k}\\ {}^{h}v{}_{k} \end{array}\right]$$

as done, e.g., in [22,24]. The measurement equations are nonlinear, which forces to compute their Jacobian matrices when carrying out the linearization process; however, the computations are neither algebraically difficult nor computationally demanding. The derivation of the measurement noise covariance matrix is not difficult as well, since it can be expressed directly in terms of the statistics of the measurement noise affecting each sensor.

### 5.4. Vector Selection

Another important issue in the EKF design deals with its behaviour when anomalous measurements are received. It takes relatively long for an EKF to recover from false measurements when they are given the opportunity to contribute to the estimate of the state vector [57]. The best approach to deal with this problem would consist of preventing the filter from processing data whose reliability is suspected to be low [22,24].

As for the acceleration, in order to answer the question whether the body-fixed measured acceleration vector is suitable for measuring gravity, we would compare its norm with the known value of gravity; better yet, we may decide to work directly with the norm of the difference between the measured acceleration vector, resolved in the earth-fixed frame, and the gravity. If the deviation exceeds some properly chosen threshold value, a sensor glitch, or a contamination due to body motion would be suspected. Different actions can be taken: the measured vector is discarded, and the filter update is only based on magnetic measurements, unless they too are considered unreliable. This is equivalent to temporarily set the acceleration measurement noise variance to some large value, which can be considered, to all practical purposes, infinite:

(70) $${}^{R}\sigma_{a}^{2}=\{\begin{array}{cc} \sigma_{a}^{2}, & \left|a_{k}-{}_{E}^{B}C\;\left({\bar{q}}_{k-1}^{-}\right)\;g\right|<{ɛ}_{a}\\ \infty , & \text{otherwise}, \end{array}$$

where *ɛ_a_* is a suitably chosen threshold. An alternative approach consists of defining a suitable law for relating the increase of the acceleration measurement noise variance to the actual deviation of the measured acceleration vector from the gravity. According to our experience, the norm-based adaptive algorithm (70) works adequately in most practical conditions. Note that the quaternion predicted by the filter at time *t*_*k*−1_ is used in (70), in place of the unknown true quaternion at the time instant *t_k_*.

A similar approach can be pursued as for the magnetic measurements. In this case we have to work with the difference between the measured magnetic vector, resolved in the earth-fixed frame, and the local magnetic reference vector. Sometimes, computing the dip angle is suggested to help improving the process; the dip angle is the angle between the magnetic field and the horizontal plane, which would be constant for given latitude and longitude. Unfortunately, the dip angle varies very erratically, especially within indoor environments, and we find its use somewhat critical. An effective norm-based adaptive algorithm applied to the magnetic sensor measurement noise variance is as follows:

(71) $${}^{R}\sigma_{h}^{2}=\{\begin{array}{l} \sigma_{h}^{2},\left|h_{k}-{}_{B}^{E}C\;\left({\bar{q}}_{k-1}^{-}\right)\;h_{\text{earth}}-{}^{h}b{}_{k-1}^{-}\right|<{ɛ}_{h}\\ \infty ,\text{otherwise}, \end{array}$$

where *ɛ_h_* is a suitably chosen threshold. The problem with this approach is that we must assume that the local magnetic reference vector is known, which is not the case unless the magnetic bias auto-calibration feature is implemented in the EKF. Note that the magnetic bias predicted by the filter at time *t*_*k*−1_ is used in (71), in place of its unknown true value at time *t_k_*.

A final comment concerns the applications in gait analysis, in the particularly important case that the IMMU is placed on the lower limbs, e.g., on the foot instep [65]. Rather than fusing them in an EKF, accelerometer data are used just to align the IMMU when the foot is at rest (stance phase of the gait cycle), followed by gyro integration during the swing phase of the gait cycle. During the swing phase the acceleration signals are indeed dominated by the inertial component, which means that the condition implied by (70) is almost never fulfilled. The stride-by-stride gyro integration reset allows mitigating the random-walk errors associated with the integration of gyro wideband measurement noise. To perform stance detection, we need a variant of the norm-based adaptive algorithm (70), or possibly a similar algorithm applied to gyro data:

(72) $$\left|a_{k}-{}_{E}^{B}C\;\left({\bar{q}}_{i-1}^{\;-}\right)\;g\right|<{ɛ}_{a}\;\cup \;\;\left|\omega_{k}\right|<{ɛ}_{g}\;\text{for}\;i\in \left[k-K,k-1\right],$$

where *ɛ_g_* is a suitably chosen threshold, and *KT_s_* is the given amount of time to detect when the foot is at rest.

### 5.5. Filter Parameter Tuning

The parameters to be tuned in an EKF concern the statistical properties assumed for the process noise and the measurement noise. Since they are modelled as Gaussian noise, we need to specify the possibly time-variant covariance matrices **R***_k_* and **Q***_k_* [57,69].

The measurement noise covariance matrix **R***_k_* is usually built from an isotropic model of sensor behaviour: the sensing elements that form each triad are characterized by the same measurement noise variance, hence **R***_k_* is proportional to a identity matrix, or it presents a block-diagonal structure. The measurement noise variances are estimated by taking samples from the sensors at a stationary location. The estimated measurement noise variances can be slightly increased over the on-bench calibration values, to help the EKF stability. In this way, for instance, tremulous motions that an accelerometer can be subject to or minute magnetic variations that can be observed for even very small sensor displacements can be accounted for as noise components in the filter. The EKF is generally quite robust to mismodelling errors in the measurement noise covariance matrix.

Once the structure of the process noise covariance matrix **Q***_k_* is determined, it can be convenient to use scaling parameters that are applied to specific blocks of **Q***_k_*. The values of the scaling parameters can be determined using a non-linear optimization routine for values that optimize the filter behaviour in given operating conditions [59]. Some considerations in parameter tuning must be directed to the belief attached to the validity of the process model. In assessing the model validity, for instance, we have to consider the many gyro error sources besides bias that are not modelled using (68), and the assumption that the angular velocity is constant in the time interval between consecutives updates by the EKF. In particular, the latter assumption can be criticized either for high dynamic motions, low sampling frequencies or both. In any case, it is known that increasing the value of the scaling parameters tends to make the filter response more prompt, at the expense of some degradation in the accuracy of the state vector estimates when the system’s behaviour is more benign. The filter response can also be adjusted by on-line adaptation of **Q***_k_*. This can be done according to different means, e.g., by detecting statistically significant changes in the innovation produced by the filter—fading memory algorithm, [70]. We do not introduce any **Q**-adaptation in the exemplary EKF presented in this paper. In general, it would be said that the EKF is less robust to mismodelling errors in **Q***_k_* than in **R***_k_*.

A study concerning the EKF performance assessment can be performed on experimental or synthetic motion data. With the former approach a series of experiments is performed, where monitored subjects are asked to perform paradigmatic motions. The main problem is that the true motion signals are unknown, because of the noise present in the measurement data. It is possible to smooth the data to some extent and use them as a reference signal, but the risk is to introduce false signal features, while removing true ones. With the latter approach, the main problem is whether the synthetic signals really capture the exact characteristics of the paradigmatic motions or not. If not, it is difficult to predict filter performance when applied to real-life tasks. As neither of these approaches is perfect, both analyses are usually performed and attempts are made to relate them to each other.

Mostly, in order to construct a dataset according to the approach based on experimental motion data, the motion is recorded using a gold standard optical tracker. The truth-reference unit quaternion **q̄**_true_ can be built from the 3D-position coordinates of a minimum of three markers, using, e.g., the Horn algorithm [71]. Quaternion smoothing can be performed by using norm-preserving orientation filters [72] or by independently filtering the quaternion components with any standard low-pass filter, followed by “brute force” normalization [22]. In order to implement a simulation environment for Monte Carlo simulation studies, standard conversion formulas can be applied to construct the orientation vector from the unit quaternion [30]. The orientation vector and its time derivative are then used to synthesize the angular velocity vector that generates the specified orientation [73]. The sensed gravity and magnetic field are computed from resolving gravity and magnetic field into the body frame using (32); additive white Gaussian noises with null mean and assigned variance are added to simulate the sensor measurements during the motion. Any given disturbance, e.g., body acceleration and magnetic variation can also be added into the simulated sensor signals before resolving them in the body-fixed frame.

### 5.6. Filter Performance Assessment

The performance metrics can be based on computing $\Delta \bar{q}={\bar{q}}_{\text{true}}^{\;-1}⊗\bar{q}$, where **q̄**_true_ and **q̄** are the true and the estimated quaternions, respectively. The quaternion Δ**q̄** represents therefore the rotation that brings the estimated body frame onto the true body frame. The orientation error Δ*θ* is obtained from the scalar component of Δ**q̄** according to the equation Δ*θ* = 2 arccos (Δ*q*_4_). The performance metrics are expressed in terms of the root-mean-square-value of the orientation error (RMSE*_θ_*), averaged over the number of either the Monte Carlo simulation runs or the experimental trials available. Alternatively, a set of estimated and reference Euler angles can be computed from **q̄**_true_ and **q̄** using standard conversion formulas, and the filter performance can be summarized by presenting the RMSEs of the Euler angles, again averaged over the number of either the Monte Carlo simulation runs or the experimental trials available. An obvious advantage of working with synthetic motion signals is that the errors incurred in estimating the state vector components can be compared with the bounds that are predicted by the error covariance matrix produced by the EKF. This is a useful feature to assess the filter convergence and to diagnose a number of potential problems arising in its numerical implementation. Of course, this possibility is precluded when working with experimental motion signals.

### 5.7. Exemplary Direct-State EKF

The dynamic model equation is as follows:

(73) $$\left[\begin{array}{c} {\bar{q}}_{k}\\ {}^{h}b_{k} \end{array}\right]=\left[\begin{array}{cc} \text{exp}\;\left[\Omega \;\left(\omega_{B}\;\left(t_{k-1}\right)\right)T_{s}\right] & 0\\ 0 & I_{3} \end{array}\right]\;\left[\begin{array}{c} {\bar{q}}_{k-1}\\ {}^{h}b_{k-1} \end{array}\right]+[\begin{array}{c} {}^{q}w_{k-1}\\ {}^{h}w_{k-1} \end{array}]\;.$$

The dynamic model is linear and time-variant. Implicit in the formulation (73) is that the gyro biases are negligible. The auto-calibration feature implemented in the filter is limited to handling the problem of magnetic variations, by modelling the magnetic bias as a random-walk process driven by the zero-mean white Gaussian noise vector *^h^***w***_k_*.

The validity of the model part that describes the time-evolution of the unit quaternion depends on the assumption that the angular velocity is constant in the time interval [*t*_*k*−1_ *t_k_*]. The process noise component is:

(74) $${}^{q}w_{k-1}=-\frac{T_{s}}{2}\;\Xi \;\left({\bar{q}}_{k-1}\right){}^{g}\;v_{k-1}=-\frac{T_{s}}{2}[\begin{array}{c} \left[q_{k-1}\times \right]+q_{4\;(k-1)}I_{3}\\ -q_{k-1}^{T} \end{array}]\;.$$

*^q^***w**_*k*−1_ describes how the gyro noise enters the state model through a quaternion-dependent linear transformation. The process noise component *^h^***w**_*k*−1_ is assumed to have covariance matrix ${}^{b}\Sigma =T_{s}\;\sigma_{b}^{2}\;I_{3}$. The variance term $\sigma_{b}^{2}$ reflects the *a priori* belief about the severity of the magnetic variations in the given environment.

Because of the assumption that *^q^***w**_*k*−1_ and *^h^***w**_*k*−1_ are uncorrelated, the process noise covariance matrix **Q***_k_* has the following block-diagonal structure:

(75) $$Q_{k-1}=[\begin{array}{cc} {\left(\frac{T_{s}}{2}\right)}^{2}\Xi_{k-1}{}^{g}\Sigma \Xi_{k-1}^{T} & 0\\ 0 & {}^{b}\Sigma \end{array}]\;.$$

Equation (69) describes the measurement model. The measurement noise covariance matrix is:

(76) $$R_{k}=[\begin{array}{cc} {}^{a}R_{k} & 0\\ 0 & {}^{h}R_{k} \end{array}]\;,$$

with:

(77) $$\{\begin{array}{c} {}^{a}R_{k}={}^{R}\sigma_{a}^{2}\;I_{3}\;\\ {}^{h}R_{k}={}^{R}\sigma_{h}^{2}\;I_{3}. \end{array}$$

where the variance terms are determined using (70)–(71) (**R**-adaptation). Useless to say, when both variance terms are set to some extremely large values, *i.e.*, no aiding comes to the filter, the kinematic equations are integrated based only on the gyro data.

**Figure 6.**
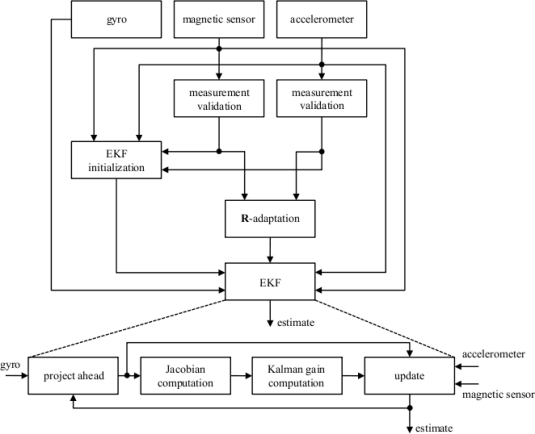
EKF structure.

The block diagram of the filter is sketched in Figure 6. The block “project ahead” computes the *a priori* state estimate and error covariance matrix, using (A2)–(A3). The estimation of the rotation matrix is carried within this block; this estimate is also used to compute the Jacobian matrix of the measurement Equation (69), using (A10), and the Kalman Gain, using (A5)–(A6). The block “update” computes the *a posteriori* state estimate and error covariance matrix, using (A7)–(A8). Remind the need for normalizing the updated quaternion at this level, in preparation for the next “project ahead” step. The measurement validation tests implement (70)–(71), which is followed by the **R**-adaptation step, via (76)–(77). The iterative nature of the filter allows exploiting the statistics available at any time step to start the computations at the next time-step, when a new set of measurements from the sensors become available.

### 5.8. Head Motion Tracking Trial

The dataset for the experiment described in this Section was obtained by collecting inertial/magnetic sensor data from the MTx orientation tracker by Xsens Technologies B.V., Enschede, The Netherlands. These data were delivered through the USB interface to a host computer at a rate of 100 Hz together with the unit quaternion time functions estimated by the native Xsens EKF that runs in its default setting. The device was placed on top of a 10 cm × 10 cm plate that was screwed on a cyclist helmet, and fastened using double-side adhesive tape. The plate orientation was recorded using a six-camera Vicon optical tracker with a sampling rate of 100 Hz. A trigger signal was generated by the host computer and enabled time-synchronization of MTx and Vicon data streams. The system measured the position of four reflective markers (diameter: 25 mm), placed at the corners of the plate.

The MTx sensors were calibrated before starting the experimental session and their scale factor and bias errors were therefore zeroed for all practical purposes. The initial orientation of the sensor frame relative to the reference frame was found by asking the subject to stand still for few seconds at the beginning of the trial. The calibration quaternion needed to estimate the reference magnetic vector was built during the still time. The subject was then asked to freely move and turn his helmet-capped head during the trial, while standing on the spot. The head motion trial lasted slightly more than half a minute. The Euler angles time functions delivered by the Vicon system were considered the truth reference for the purpose of error estimation.

The EKF was implemented in Matlab for off-line data processing on a MacBook Air computer. Using the virtualization technology from Parallels Desktop 4.0 for Mac, the cycle time for a single iteration turned out to be about 2.0 ms, without any particular programming effort made to optimize the computational efficiency of the filter. The optimally tuned parameter setting for the EKF is reported in Table 1.

The error statistics are reported in Table 2.

The first and last column report the errors incurred by the EKF and the Xsens EKF, respectively. The columns 3 to 5 give the errors incurred when the gyro is aided by: accelerometer only (column 3); magnetic sensor only (column 4); no sensors (column 5). Suppose that (71) is implemented with *ɛ_h_* = 0 The measurements from the magnetic sensor cannot be incorporated in the measurement update stage of the EKF; the same occurs when *ɛ_a_* = 0, as for the accelerometer, in (70). When both thresholds are zero, the gyro-free aiding sensor system is inhibited, and the orientation solution is obtained entirely from gyro measurement data. It is apparent that the accelerometer and the magnetic sensor are helpful in inclination and heading stabilization, respectively. Without aiding sensors, random-walk integration of gyro wideband measurement noise yields seriously degraded performance. These results clearly indicate the importance of sensor fusion in improving the accuracy of orientation estimates by inertial/magnetic sensing.

**Figure 7.**
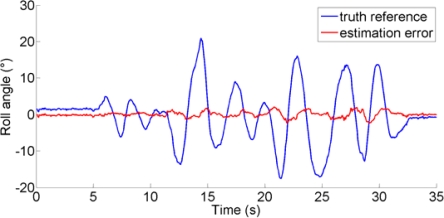
Roll angle time functions, truth reference and estimation error by the EKF.

**Figure 8.**
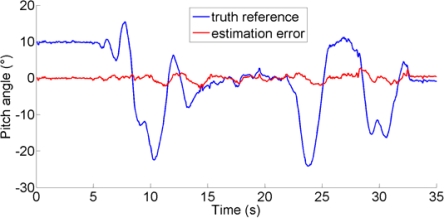
Pitch angle time functions, truth reference and estimation error by the EKF.

**Figure 9.**
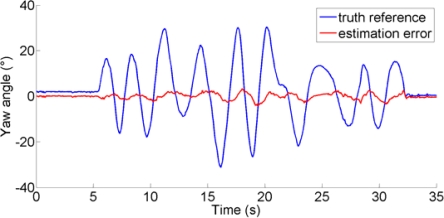
Yaw angle time functions, truth reference and estimation error by the EKF.

Finally, Figures 7–9 show the time functions of the Euler angles as they are measured from the Vicon system; superimposed on them the time functions of the estimation errors incurred by the EKF.

## 6. Concluding Remarks

A comprehensive review of the endless literature on the problem of orientation determination is virtually impossible and necessarily incomplete, especially if one wishes to encompass all possible applications. We hope this article is interesting to experts and novice alike; the reported information would be sufficient to the readers, in order to cook their formulation of a state-of-the-art algorithm for 3D-orientation estimation using inertial/magnetic sensing in applications of human body motion tracking and analysis.

## Figures and Tables

**Table 1.** Optimally tuned parameter setting for the EKF. The raw magnetic data from the MTx are expressed in arbitrary units (a.u.), since they are normalized to earth field strength by the manufacturer.

**Process noise statistics**	
Gyro standard deviation *^g^σ*, °/s	0.4
Magnetic bias standard deviation *^b^σ*, a.u. (× 10^−3^)	0.1
**Measurement noise statistics**	
Accelerometer standard deviation *^a^σ*, m*g*	10.0
Magnetic sensor standard deviation *^h^σ*, a.u. (× 10^−3^)	1.0
**Thresholds for R-adaptation**	
Acceleration measurements: *ɛ*_*a*_, m*g*	40.0
Magnetic sensor measurements: *ɛ*_*h*_, a.u. (× 10^−3^)	50.0

**Table 2.** Performance assessment.

RMSE	EKF	*ɛ_h_* = 0	*ɛ_a_* = 0	*ɛ_a_* = *ɛ*_*h*_ = 0	Xsens EKF
Roll angle, °	0.72	0.89	3.13	0.97	0.94
Pitch angle, °	0.83	0.88	1.20	4.91	0.76
Yaw angle, °	1.23	3.88	4.30	3.76	1.30
Orientation angle, °	1.62	3.96	5.26	6.19	1.72

## Appendix

In this Appendix we report in short the equatons of both the KF and the EKF. For an excellent treatment of these topics, consult [57].

### 1. KF Equations

Dynamic and measurement models:

(A1)
$$\begin{array}{l} x_{k}=F_{k-1}x_{k-1}+w_{k-1}\\ z_{k}=H_{k}x_{k}+v_{k}; \end{array}$$

1. Compute the *a priori* state estimate:
  (A2)
  $$x_{k}^{-}=F_{k-1}x_{k-1}^{+};$$
  the superscript—in $x_{k}^{-}$ stands for “the *a priori* estimate at time *t_k_*, before the current measurement **z***_k_* is used in computing the *a posteriori* estimate”; the superscript + in $x_{k-1}^{+}$ stands for “the *a posteriori* estimate at time *t_k_*, which is computed based on the evidence in the current measurement **z***_k_*”.
2. Compute the a priori error covariance matrix:
  (A3)
  $$P_{k}^{-}=F_{k-1}P_{k-1}F_{k-1}^{T}+Q_{k-1};$$
3. Compute the innovation and its covariance matrix:
  (A4)
  $$\nu_{k}=z_{k}-H_{k}\;\left(x_{k}^{-}\right);$$
4. Compute the covariance matrix of the innovation:
  (A5)
  $$S_{k}=H_{k}P_{k}^{-}H_{k}^{T}+R_{k};$$
5. Compute the Kalman gain:
  (A6)
  $$K_{k}=P_{k}^{-}H_{k}^{T}S_{k}^{-1};$$
6. Compute the *a posteriori* state estimate:
  (A7)
  $$x_{k}^{+}=x_{k}^{-}+K_{k}\nu_{k};$$
7. Compute the *a posteriori* error covariance matrix:
  (A8)
  $$P_{k}^{+}=P_{k}^{-}-K_{k}H_{k}P_{k}^{-}.$$

### 2. EKF Equations

When the dynamic model, the measurement model or both are nonlinear the EKF comes to our rescue:

(A9)
$$\begin{array}{l} x_{k}=f_{k-1}\;\left(x_{k-1}\right)+w_{k-1}\\ z_{k}=h_{k}\;\left(x_{k}\right)+v_{k}. \end{array}$$

The EKF equations differ from the Equations (A1)–(A8) only in the specifications of the matrices **F**_*k*−1_ and **H***_k_*:

(A10)
$$\{\begin{array}{c} F_{\mathrm{ijk}-1}={\frac{\partial }{{\partial x}_{j}}f_{i}\;\left(x\right)|}_{x=x_{k-1}^{+}}\\ H_{\mathrm{ijk}}={\frac{\partial }{{\partial x}_{j}}h_{i}\;\left(x\right)|}_{x=x_{k}^{-}.} \end{array}$$
